# Oral Microcosm Biofilms Grown under Conditions Progressing from Peri-Implant Health, Peri-Implant Mucositis, and Peri-Implantitis

**DOI:** 10.3390/ijerph192114088

**Published:** 2022-10-28

**Authors:** Vanessa Sousa, Dave Spratt, Mehmet Davrandi, Nikos Mardas, Víctor Beltrán, Nikolaos Donos

**Affiliations:** 1 Periodontology and Periodontal Medicine, Centre for Host-Microbiome Interactions, Faculty of Dentistry, Oral and Craniofacial Sciences, Kings College London, Guy’s and St Thomas’ NHS Foundation Trust, London SE1 9RT, UK; 2 Microbial Diseases, Eastman Dental Institute, University College London, London WC1E 6BT, UK; 3 Centre for Oral Clinical Research, Centre for Oral Immunobiology and Regenerative Medicine, Institute of Dentistry, Barts and The London School of Medicine and Dentistry, Queen Mary University London, London E1 2AD, UK; 4 Clinical Investigation and Dental Innovation Center (CIDIC), Dental School and Center for Translational Medicine (CEMT-BIOREN), Universidad de La Frontera, Temuco 4780000, Chile

**Keywords:** peri-implantitis, therapy, microcosm, oral biofilm, modelling biofilms, microbiome, microbial ecology

## Abstract

Peri-implantitis is a disease influenced by dysbiotic microbial communities that play a role in the short- and long-term outcomes of its clinical treatment. The ecological triggers that establish the progression from peri-implant mucositis to peri-implantitis remain unknown. This investigation describes the development of a novel in vitro microcosm biofilm model. Biofilms were grown over 30 days over machined titanium discs in a constant depth film fermentor (CDFF), which was inoculated (I) with pooled human saliva. Following longitudinal biofilm sampling across peri-implant health (PH), peri-implant mucositis (PM), and peri-implantitis (PI) conditions, the characterisation of the biofilms was performed. The biofilm analyses included imaging by confocal laser scanning microscopy (CLSM) and scanning electron microscopy (SEM), selective and non-selective culture media of viable biofilms, and 16S rRNA gene amplification and sequencing. Bacterial qualitative shifts were observed by CLSM and SEM across conditions, which were defined by characteristic phenotypes. A total of 9 phyla, 83 genera, and 156 species were identified throughout the experiment. The phyla *Proteobacteria*, *Bacteroidetes*, *Firmicutes*, *Fusobacteria*, and *Actinobacteria* showed the highest prevalence in PI conditions. This novel in vitro microcosm model provides a high-throughput alternative for growing microcosm biofilms resembling an in vitro progression from PH–PM–PI conditions.

## 1. Introduction

Dental implants have become increasingly popular treatment choices; by 2026, implant prevalence is expected to increase to 23% among adults [1]. However, the prevalence of biological complications around dental implants continues to increase as well [2]. Peri-implant diseases are clinical realities in everyday practices, with approximately 1/3 of patients (and 1/5 of all implants) experiencing peri-implantitis [3]. Importantly, the microbial shifts for the progression from peri-implant mucositis to peri-implantitis are poorly understood [4,5]; whilst different solutions have been advocated for the treatment of peri-implantitis, at present there is no gold standard therapy that can predictably resolve this condition [6].

There are a number of in situ and in vitro methods for the investigation of oral biofilms [7,8,9,10,11,12]. However, to date, no suitable in vitro models [13] have been developed that enable the replication of the complex microbial composition of peri-implantitis to allow testing and development of new clinically-relevant therapies [14], which can shed light on the characterisation and understanding of processes and factors affecting biofilms. Even though every model has limitations and cannot reproduce all the details involved in the formation of either dental and/or implant biofilms, by performing reproducible experiments in controlled environments that closely mimic the characteristics found in the oral cavity *in vivo*, these models can aid in addressing key research questions, for instance, to acquire information and to clarify the results and outcomes of a given therapy [10,15,16].

A number of dental biofilm model systems, often referred to as artificial mouth models (AMM), have been developed over the last three decades [17]. These AMMs allow for the control of multiple environmental factors based on clinical parameters found in the patient’s mouth, thus allowing for the detailed analysis of several aspects of biofilm formation and characterisation.

The constant depth film fermentor (CDFF), a continuous open-surface fluid flow culture AMM, is one of the most widely used in vitro oral model systems for the evaluation of oral biofilms and therapeutic strategies [10,17,18,19]. Its relevance to the oral cavity mainly relies on (1) the use of an inoculum derived from human whole saliva, (2) the mimicking continuous nutrient flow conditions of the salivary and crevicular fluid flow, (3) the shear forces induced to the biofilms by the PTFE scraper blades, (4) the biofilms can be grown on a range of substrata, and (5) the induction of a range of atmospheric environments [11]. The steady-state microcosm biofilms can be sampled and qualitatively and quantitatively analysed.

The aim of this investigation was to develop a novel in vitro microcosm biofilm model to analyse steady-state biofilm communities associated with shifts, progressing from health to disease and, thus, emulating peri-implant health, peri-implant mucositis, and peri-implantitis conditions over titanium substrata.

## 2. Materials and Methods

### 2.1. Selection of Donors

Ethical approval of the protocol for human whole saliva sample collection and experimental research was provided by UCL Research Ethics Committee approval (1364/001). Whole saliva was collected from 37 healthy donors who were >18 years old. Participants were excluded if they had used antibiotics or used antimicrobial mouthwash during the last 3 months. Prior to the sampling, patients refrained from consuming alcohol, brushing their teeth, or consuming food. Sampling was performed according to a standardised procedure. Unstimulated saliva was collected using the spitting method [20] for a total of 5 min per donor. Equal amounts of unstimulated whole saliva from each donor were pooled along with glycerol (final concentration 15%, [*v*/*v*]). After homogenising the sample, 1 mL aliquots were stored at −80 °C for later processing for sequencing analysis of inoculum composition and inoculation of the CDFF.

### 2.2. Microcosm Biofilm Model

Biofilms were grown in a CDFF bioreactor [21]. The resemblance (stability and reproducibility) of this system was tested by performing two independent CDFF runs, following protocols previously established [10,22]. To summarise, biofilms were formed directly on titanium (Ti) surfaces placed on top of the PTFE plugs of the sample pan (5 Ti discs per pan). Specifically, commercially pure machined (S_a_ = 0.40 µm) Ti discs (grade IV; ASTM F 67; Institut Straumann AG, Basel, Switzerland) of 5 mm in diameter and 1 mm in thickness were used. To provide nutrients and atmospheric conditions associated with peri-implantitis, a CDFF run was designed to emulate the conditions associated with the development of a peri-implantitis microcosm biofilm for 30 days.

The CDFF was inoculated with 500 mL of artificial saliva [10,21] containing a 1 mL aliquot of a pooled stock of human whole saliva. Once inoculated, the sterile artificial saliva growth media was pumped into the CDFF at a rate of 0.5 mL/min up to day 19 [18].

A formulation of artificial tissue fluid, mimicking in vitro the peri-implant sulcular fluid (PISF) based on 60% RPMI tissue culture medium, supplemented with 40% horse serum, 0.5 µg/mL of menadione, and 5 µg/mL of haemin was pumped into the bioreactor. The PISF formulation flowed into the CDFF at a rate of 40 µL/min during healthy and peri-implant mucositis conditions. The rate increased to 130 µL/min on day 9, which was accompanied by a switch to a microaerophilic environment by pumping a microaerophilic gas mixture (2% O_2_, 3% CO_2_, 95% N) at a rate of 200 cm^3^/min with an oxygen content associated with peri-implant mucositis [23]. Finally, on day 20, an anaerobic environment (5% CO_2_, 95% N at 200 bar) emulating peri-implantitis conditions was performed. The microenvironments were induced via a filtered air inlet at the top of the plate of the CDFF [24].

### 2.3. Harvesting of Microcosm Biofilms

The steady-state biofilms were obtained during the course of the run at health (days 2, 4), peri-implant mucositis (days 9, 10, 16, and 19), and peri-implantitis (days 21, 22, 25, 26, and 30) conditions. Microcosm biofilms were aseptically removed throughout the study period from healthy to peri-implantitis conditions. The sampled discs were placed into an appropriate vessel depending on the type of analysis (e.g., imaging or microbiological characterisation) to be performed.

### 2.4. Characterisation by Culture Media Analyses

Selective and non-selective culture media analyses of fresh biofilms, 2 discs per experiment (*n* = 4), and duplicate plates with duplicate aliquots (*n* = 8) were performed by the sampling point and inoculum.

The biofilms were aseptically removed from the CDFF and placed in 1 mL of sterile reduced transport fluid (RTF) containing five glass beads and then vortexed for 1 min to disrupt the biofilm. Aerobes were cultured by plating the dilutions onto Columbia Blood Agar (CBA, LabM, Lancashire, UK). Anaerobes were isolated to Fastidious Anaerobe Agar (FAA, LabM, Lancashire, UK). *Streptococcus* spp. were selected on Mitis Salivarius Agar (Sigma-Aldrich, Dorset, UK), and cadmium fluoride–acriflavine–tellurite agar (CFAT) (14) was used for selecting *Actinomyces* spp. *Staphylococcus* spp. were selected on mannitol salt agar (Oxoid, Hampshire, UK). Gram-negative (G-) species were selected on Fastidious Anaerobe Agar with a supplement of 5 ug/mL of vancomycin hydrochloride. Sabouraud Dextrose Agar (Oxoid, Hampshire, UK) was used for the isolation of *Candida* spp., and *Veillonella* agar (BD Biosciences, Wokingham, UK) for the isolation of *Veillonella* spp. Inoculated plates were incubated as appropriate either aerobically (5% CO_2_) at 37 °C overnight, or in an anaerobic atmosphere (5% CO_2_, 5% H, 90% N) at 37 °C for 4 days. Colonies were counted and CFU/mL/biofilm was calculated. On selective media, species were confirmed by colony morphology and Gram staining.

### 2.5. Characterisation by 16S rRNA Amplification and Sequencing

The 16S rRNA amplification and sequencing (Illumina Inc., MiSeq, San Diego, CA, USA), 1 disc per experiment, and duplicate runs, including the inoculum and sampling points per condition (PH: 2, 4; PM: 9, 10, 19; PI: 22, 26, 30) (*n* = 18), was performed.

DNA was extracted and then independent PCR reactions were performed for each sample, to amplify the V5–V7 hypervariable regions with barcoded primers 785F (F 5′-GGATTAGATACCCBRGTAGTC-3′) and 1175R (R 5′-ACGTCRTCCCCDCCTTCCTC-3′) (Sigma, Gillingham, UK). Barcoded primers allow for multiplexing into one amplicon library. The PCR mixtures contained 2.5 µL of 1X Molzym PCR Buffer (Molzym GmbH & Co. KG, Bremen, Germany), 0.5 µL dNTPs (10 mM stock, Bioline, London, UK), 1 µL of each primer (10 µM stock), 0.125 µL of Moltaq (0.025 µM, VH Bio, Gateshead, UK), 0.25 µL of MgCl_2_ (50 mM stock), and approximately 100 ng of DNA template in a final volume of 25 µL (PCR grade water, Bioline, London, UK). The PCR conditions were 95 °C for 5 min, 30 cycles of 94 °C for 30 s; 55 °C for 40 s; and 72 °C for 90 s extension; followed by 72 °C for 10 min. Following the manufacturer’s instructions, the PCR products were quantified (Qubit^®^ HS DNA kit, Life Technologies, Waltham, MA, USA), purified (High Stringency Agencourt AMPure XP, Beckman Coulter Ltd., High Wycombe, UK), and combined in equimolar ratios (EB Buffer, Qiagen, Manchester, UK), to create a DNA pool for sequencing. The 16S rRNA gene fragments were sequenced using synthesis chemistry for bidirectional amplicon sequencing (MiSeq Desktop Sequencer; Reagent Kit v2; Illumina Inc., San Diego, CA, USA).

### 2.6. Imaging Microcosm Biofilms

Imaging analyses confocal laser scanning microscopy (CLSM) and scanning electron microscopy (SEM) were performed in 2 discs per experiment (*n* = 4) and triplicate areas (*n* = 12).

Selected biofilms at specific time points (as described in the publication) were stained (Live/Dead BacLight™, Life Technologies, Carlsbad, CA, USA) and imaged under CLSM (Leica SPE confocal) (*n* = 3) at different random locations. They were visualised under an upright Leica SPE confocal operated in sequential mode (excitation PMT1 488 nm (to excite the SYTO^®^9 dye) and 532 nm (to excite the propidium iodide dye)) with an objective HCX APO L U-V-I 40.0 × 0.80 W. Two-channel confocal image stacks were collected in a standardised way. The scanner settings and x–y–z dimensions were as follows: step size 0.80 µm, voxel-height 244 nm, voxel-depth 244 nm, voxel-volume 797.3 nm, zoom 1.5, pinhole (m) 141.5 um, 1024 × 1024 pixels/inch, and 63 z-slices. Images were collated and the reconstruction of the RGB stacks allowed for the analysis of the spatial dimensions (e.g., orthogonal slices and oblique slices of the biofilms). The ISO surface volume (µm^3^) rendering and analyses of all the biofilms were performed using IMARISx64 v.8.0.1 (Bitplane Inc., Abingdon, UK) software.

SEM images were taken from biofilms to study the morphological characteristics of the bacteria. Briefly, biofilms were carefully submerged in ultrapure high-quality water (UHQ water) to remove any unattached or loosely associated bacteria. Then the suspension was removed from the wells and replaced with 4% glutaraldehyde with 0.1 M cacodylate buffer (Agar Scientific, Stansted, Essex, UK) for 24 h at 4 °C. Afterwards, the glutaraldehyde fixative was replaced with a graded series of ethanol (EtOH) solutions at the concentrations of 20%, 50%, 70%, 90%, and 100% (3 times) for 10 min each to dehydrate the specimens. The final concentration of EtOH was replaced with hexamethyldisilazane (HMDS; TAAB Ltd., Aldermaston, UK) for 2 min. The specimens were then left to dry overnight in a desiccator. The discs were mounted onto individual aluminium SEM specimen stubs (Agar Scientific, Stansted, Essex, UK) and sputter-coated with gold/palladium by means of a Polaron E5100 coating device (Polaron CVT, Quorum Technology, Lewes, UK). The analysis of the discs was done in triplicates using a SEM JEOL JSM 5410LV (JEOL UK, Welwyn Garden City, UK) using various magnifications at an operating voltage of 10 kV.

### 2.7. Sequencing Data Analysis

Sequencing data were processed using QIIME 2 (Version 2018.11, https://qiime2.org/ (accessed on 01 August 2019)) [25]. Raw sequences belonging to 18 samples were de-multiplexed with q2-demux and then denoised using DADA2 plugin with default parameters to create amplicon sequence variants (ASVs) [26,27]. Alpha diversity metrics were calculated on the unrarefied feature table using the native q2-diversity plugin and included a number of ASVs, the Shannon index, and Faith’s phylogenetic diversity. For beta diversity, the feature table was rarefied at 2477 sequences and used to calculate Aitchison (compositional) and weighted UniFrac (quantitative, accounts for phylogenetic information) distances [28]. These distance matrices were then fed to the principal coordinate analysis (PCoA) to investigate clustering patterns based on the metadata groupings. A taxonomic classifier optimised for the V5–V7 regions of the 16S rRNA gene from the extended human oral microbiome database (HOMD expanded, https://www.homd.org/ (accessed on 01 August 2019)) was trained using the q2-feature-classifier, and taxonomy was assigned using this classifier to ASVs at >85% confidence. With the unrarefied feature-table, the q2-gneiss plugin was used to determine differentially abundant taxa between healthy and diseased samples at the genus level [27]. Furthermore, the unrarefied feature table was transferred to the QIIME 1 platform to generate group-wise (health/disease, sampling point) taxonomic summaries using the summarize_taxa_through_plots.py function. The data resulting from the analyses mentioned above were exported from the QIIME 2 environment, and respective plots were generated in R v.3.5.0 using ggplot2 package v.3.1.0 [29].

## 3. Results

### 3.1. Culture Media Analysis

The composition of the inoculum by culture media analysis revealed that aerobic spp. (8.47 log_10_) were significantly higher than *Staphylococcus* (6.78 log_10_) and *Veillonella* spp. (6.99 log_10_) (*p* = 0.05). All biofilms showed growth of viable log_10_ CFU/mL anaerobic and aerobic counts drawn from the discs ranging from 6 log_10_ up to 9.5 log_10_. Under conditions emulating peri-implant mucositis, anaerobic counts rose by day 16 and remained higher until day 22. Then under conditions emulating peri-implantitis, the total anaerobic counts decreased, and by the end of the study period, no significant differences were found between them and the aerobic viable counts. In conditions emulating peri-implant health, *Streptococcus* (5.46 log_10_), *Staphylococcus* (5.68 log_10_), *Candida* (6.63 log_10_), and *Veillonella* (6.14 log_10_) spp. were present in significantly lower proportions (*p* = 0.05). It appears that during peri-implant mucositis *Actinomyces* (8.15 log_10_), G- (8.26 log_10_), and *Candida* spp. (8.57 log_10_) were present in high proportions; however, they were not significantly different from the aerobic spp. counts. During peri-implantitis conditions, G- spp. (8.09 log_10_) and *Actinomyces* (7.87 log_10_) yielded the highest counts.

### 3.2. Analysis of the 16S rRNA Gene and Comparative Sequencing

**Figure 1:**
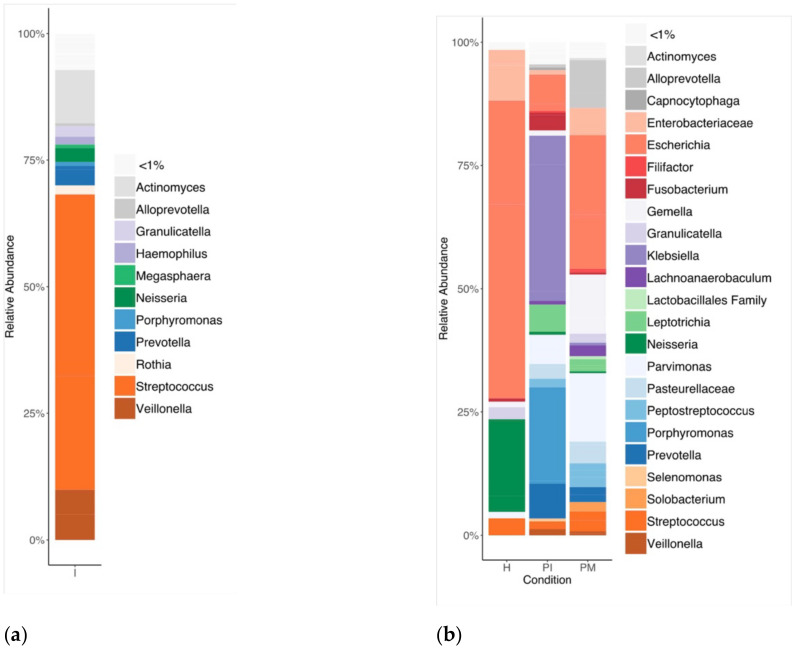
Identified bacteria genera and their relative abundances (% of total bacteria) by 16S rRNA sequencing (V5–V7). (**a**) Inoculum (I), (**b**) steady-state biofilms: peri-implant health (PH), peri-implant mucositis (PM) and peri-implantitis (PI).

**Figure 2:**
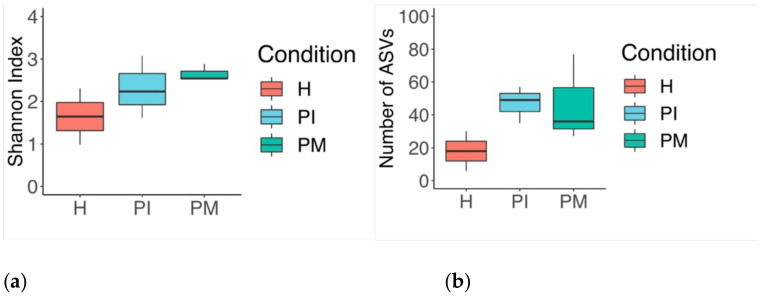
Steady-state biofilm boxplot (Tukey): (**a**) number of amplicon sequence variants (ASVs) showing the number of unique features detected in each sample, and (**b**) the Shannon index showing the degree of evenness between unique features (distributed within the community proportionality); increased richness and diversity found in the disease state.

Steady-state biofilms were grown from a whole saliva inoculum (I) and were sampled under conditions emulating peri-implant health (PH days: 2, 4), peri-implant mucositis (PM days: 9, 10, 19), and peri-implantitis (PI days: 22, 26, 30). The identified genera are represented in Figure 1 for the inoculum (I) and the relative abundances of the steady-state biofilms by condition (PH, PM, PI) (Figure 1a,b). Figure 2 shows the results from the steady-state biofilm number of amplicon sequence variants (ASVs) and the Shannon index. Shifts from health (PH) to disease (PM, PI) states were characterised by an increase in richness and diversity (Figure 2a,b).

#### 3.2.1. Phylum

Overall, a total of nine phyla were detected—Proteobacteria, Bacteroidetes, Firmicutes, Fusobacteria, Actinobacteria, Saccharibacteria (TM7), Spirochaetes, Synergistetes, and unassigned bacteria taxa.

The inoculum was primarily composed of *Firmicutes* (75.04%), *Actinobacteria* (12.62%), *Proteobacteria* (5.51%), *Bacteroidetes* (5.18%), and *Fusobacteria* (1.02%). PH conditions (days 2 and 4) were characterised by an increased abundance in *Proteobacteria* (86.71%), and *Firmicutes* (11.94%). In comparison to PH, PM conditions (days 9, 10, 19) were characterised by a reduction in *Proteobacteria* (57.38%), and an increase in the relative abundance of *Firmicutes* (11.94%), *Bacteroidetes* (16.17%), *Fusobacteria* (2.22%), and *Actinobacteria* (1.18%); whereas PI conditions (days 22, 26, 30) were characterised by a decreased relative abundance in *Proteobacteria* (49.38%), and an increase in *Bacteroidetes* (31.26%), *Firmicutes* (12.67%), *Fusobacteria* (6.38%), in comparison to PH. The presence of *Actinobacteria* (0.31%) was only observed in PI.

#### 3.2.2. Genus

A total of 83 genera were identified. The inoculum was predominately characterised by *Streptococcus* (59.97%), *Actinomyces* (10.04%), *Veillonella* (9.86%), *Prevotella* (3.50%), *Neisseria* (2.91%), *Granulicatella* (2.00%), *Rothia* (1.73%), and *Haemophilus* (1.48%). PH showed the lowest richness in *Escherichia* (53.66%), *Neisseria* (23.35%), *Enterobacteriaceae* genus (8.86%), *Streptococcus* (4.90%), *Granulicatella* (3.32%), *Parvimonas* (1.77%), and *Gemella* (1.53%). PM exhibited the highest richness, mainly composed of *Escherichia* (44.69%), *Alloprevotella* (11.85%), *Enterobacteriaceae* genus (8.19%), *Parvimonas* (6.74%), *Peptostreptococcus* (4.31%), *Prevotella* (4.19%), *Gemella* (3.53%), *Streptococcus* (2.77%), *Pasteurellaceae* genus (2.64%), *Leptotrichia* (1.65%), *Klebsiella* (1.54%), *Granulicatella* (1.26%), *Filifactor* (1.22%), *Veillonella* (1.20%), and *Actinomyces* (1.16%). PI exhibited a high bacterial richness that was higher than PH, and slightly lower than PM, comprising mainly *Klebsiella* (36.28%), *Porphyromonas* (25.30%), *Escherichia* (8.81%), *Parvimonas* (4.96%), *Prevotella* (4.86%), *Leptotrichia* (3.84%), *Fusobacterium* (2.54%), *Pasteurellaceae* genus (2.13%), *Streptococcus* (1.60%), *Peptostreptococcus* (1.44%), *Enterobacteriaceae* genus (1.40%), *Gemella* (1.15%), and *Veillonella* (1.03%).

#### 3.2.3. Sub-Analyses at the Species Level

A total of 156 species were identified; the inoculum was characterised by *Streptococcus thermophilus HMT152* (41.14%), *Streptococcus* spp. (12.59%), *Veillonella parvula HMT161* (8.94%), *Actinomyces odontolyticus HMT701* (8.48%), *Streptococcus pneumoniae HMT734* (4.53%), *Prevotella* spp. (2.77%), *Neisseria meningitidis HMT669* (2.34%), *Granulicatella adiacens HMT534* (1.79%), *Haemophilus parainfluenzae HMT718* (1.42%), *Rothia mucilaginosa HMT681* (1.36%), *Actinomyces graevenitzii HMT866* (1.26%), and *Streptococcus sanguinis HMT758* (1.20%).

PH was mainly characterised by species such as *Escherichia coli HMT574* (53.66%), *Neisseria meningitidis HMT669* (23.33%), *Enterobacteriaceae* spp. (8.86%), *Streptococcus* spp. (2.31%), *Granulicatella adiacens HMT534* (2.08%), *Granulicatella* spp. (1.25%), *Streptococcus cristatus* (1.15%), and *Parvimonas* spp. (1.04%). 

PM showed a high abundance of *Escherichia coli HMT574* (44.69%), *Alloprevotella* sp. *HMT473* (11.85%), *Enterobacteriaceae* spp. (8.19%), *Parvimonas micra HMT111* (4.33%), *Peptostreptococcus stomatis HMT112* (4.31%), *Gemella morbillorum HMT046* (3.40%), *Prevotella saccharolytica HMT781* (2.86%), *Pasteurellaceae* spp. (2.64%), *Parvimonas* spp. (2.41%), *Klebsiella pneumoniae HMT731* (1.54%), *Prevotella nanceiensis HMT299* (1.33%), *Filifactor alocis HMT539* (1.22%), *Veillonella parvula HMT161* (1.20%), *Actinomyces odontolyticus HMT701* (1.16%), and *Leptotrichia buccalis HMT563* (1.12%). 

The species observed in PI were mainly characterised by *Klebsiella pneumoniae HMT731* (36.27%), *Porphyromonas gingivalis HMT619* (25.30%), *Escherichia coli HMT574* (8.81%), *Prevotella saccharolytica HMT781* (4.67%), *Parvimonas micra HMT111* (3.85%), *Leptotrichia* spp. (3.58%), *Fusobacterium nucleatum* (2.54%), *Pasteurellaceae* spp. (2.13%), *Peptostreptococcus stomatis HMT112* (1.44%), *Enterobacteriaceae* spp. (1.40%), *Gemella morbillorum HMT046* (1.15%), *Parvimonas* spp. (1.11%), and *Veillonella parvula HMT161* (1.03%). 

### 3.3. Structural Analysis

**Figure 3:**
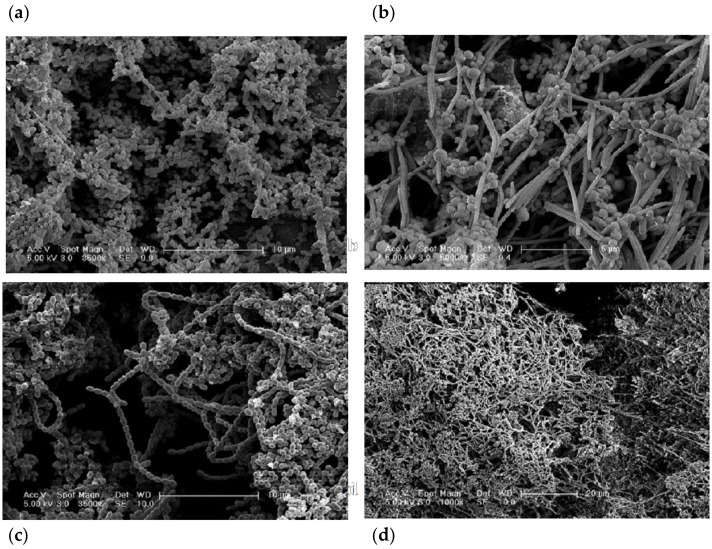
Scanning electron microscopy images showing microcosm biofilms from a CDFF 30-day peri-implantitis model. Biofilms were grown on machined Ti and sampled throughout the study period. (**a**) Represents 4-day-old biofilm grown under conditions emulating health (scale bar = 10 µm, magnification 3500×). (**b**,**c**) Represents 9- (**b**) (scale bar = 5 µm, magnification 5000×) and 15-day (**c**) biofilms after the induction of peri-implant mucositis conditions (scale bar = 10 µm, magnification 3500×). (**d**) Represents a 30-day biofilm under peri-implantitis conditions (scale bar = 20 µm, magnification 1000×).

**Figure 4:**
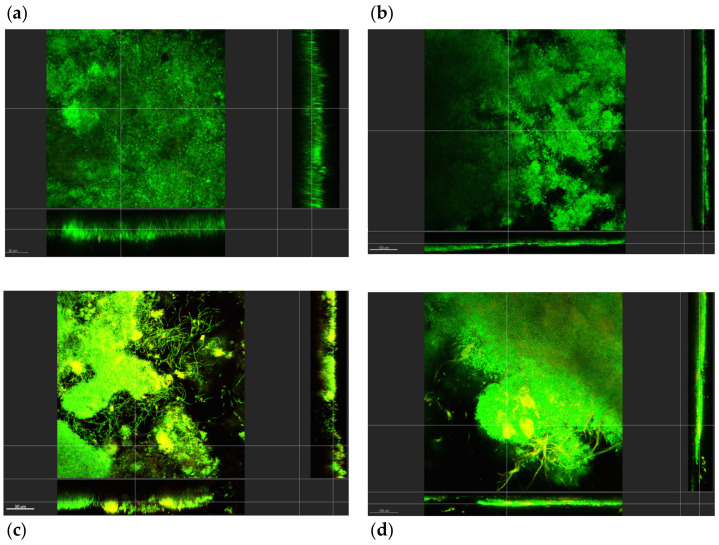
Confocal laser scanning microscopy images of microcosm biofilms grown on top of Ti surfaces at selected sampling points (4, 9, 15, and 30 days post-culture). SYTO9^®^ stained the live bacteria green while propidium iodide stained the dead bacteria red. As in the case of the SEM micrographs, the CLSM images of biofilms under health conditions on day 4 (**a**), peri-mucositis on days 9 and 11 (**b**,**c**), and peri-implantitis at 30 days (**d**) show similar bacterial growth features (x–y–z planes). The vertical view represents y–z and the sagittal view represents x–z; images were taken through layers of the z-axis.

The biofilms exhibited a composition associated with microcosm biofilms (Figure 3 and Figure 4). SEM and CLSM images of early biofilms (up to day 4) showed incomplete coverage of the implant substrata. At this early stage, the biofilm presented an open structure and consisted mainly of viable bacteria. Cocci were the predominant microbiological morphotypes under this condition (PH). A limited amount of non-viable clumps were observed.

However, as the experimental conditions progressed toward biofilm maturation, the implant surface exhibited complete coverage by microcosm biofilms. Under PM conditions on sampling days 9 and 11, apart from cocci, other bacterial morphotypes, such as rods and filaments, were present. Images showed a mature biofilm characterised by rods in chains and some filaments surrounded by cocci. Several clumps of non-viable bacteria located at random distances were noticed (Figure 3 and Figure 4). On day 30 under PI conditions, a dense and structured biofilm was observed, and the Ti surface was completely covered by extracellular polymeric substance (EPS) and large multi-layered islets of biofilms. Similar morphotypes to the peri-implant mucositis conditions, including fusiform bacteria, were noticed at this stage (Figure 3 and Figure 4). In addition, the bacterial clumps of non-viable bacteria were expressed at the maximum (Figure 3 and Figure 4); they merged/collapsed into a large structure often positioned in close contact with the Ti surface (Figure 3 and Figure 4).

## 4. Discussion

This investigation describes the development of a novel in vitro microcosm biofilm model that allows the investigation of microbial shifts in the progression of conditions resembling peri-implant health to peri-implant mucositis and peri-implantitis over 30 days. The bacterial community composition and shifts were studied in light of modifications in parameters, such as atmospheric and environmental conditions, shear forces, thickness, and nutrient source exposure. The parameters used in this study were based on previous studies from our group [22,30,31]. We further developed these in vitro conditions to resemble the peri-implant clinical conditions. The experimental conditions of this model were based on the nutrient sources (e.g., saliva and PISF) [32] and atmospheric conditions associated with *in vivo* human peri-implantitis [23,24,33,34,35,36]. In addition, a progression from health to peri-implant mucositis and finally to peri-implantitis was emulated based on previous oral biofilms CDFF-modelling literature [10,22,31]. As evidenced by the 16S rRNA sequencing results, whilst mainly facultative and aerobic species were observed under microaerophilic conditions, strict anaerobes, and G- spp. outnumbered the aerobic spp. under peri-implantitis-emulated anaerobic conditions. Similar microcosm shifts in states of health [37] and disease [22] have been described in the literature.

### 4.1. Resemblance of the Experiments

Whilst controllable and reproducible population shifts have been described in a gingivitis model [22] that employed a salivary inoculum, it has to be stated that the characterisation of bacterial profiles across shifts is difficult to perform in a microcosm CDFF system. Previous studies that employed multi-species inoculum to develop microcosm biofilms have addressed this issue [38] pointing out the reproducibility challenges arising with the use of a salivary inoculum, such as the decrease of microbial diversity due to selection processes inherent in modelling, and geographical locations of samples composing the inoculum. In order to overcome this difficulty, the same characterised inoculum was used throughout the CDFF experiments. For cryopreservation, the organisms were frozen (−80 °C) in glycerol [39]. In addition, duplicate CDFF experiments were employed.

### 4.2. Methods to Analyse and Characterise the Microcosm Biofilms

First, the growth of the bacterial community as well as the presence of contamination was assessed by the monitoring of the CDFF bioreactor, employing culture media analysis. Then, a higher bacterial resolution method, 16S rRNA sequencing, was used, in order to explore and resolve the specific composition of the microcosm.

Moreover, the configuration of these in vitro microcosm biofilms was performed by employing SEM and CLSM analyses. The aim of using these imaging techniques was to confirm the *in vivo* appearance of structures associated with biofilms in states of health and disease. Specifically, the CLSM allowed the investigation of the spatial arrangement of these three-dimensional structures. These biofilms exhibited an intricate channel system between the stacked bacterial communities. Interestingly, intercommunication between the biofilm-stacked structures was observed through bacterial species, such as filaments. However, the development of a more compact and tightly packed biofilm structure was observed in mature (e.g., peri-implantitis) biofilms. This is compatible with previous *in vivo* plaque studies of mature biofilms [40,41].

### 4.3. CDFF Modelling

Modelling microbial shifts of oral biofilms associated with health and disease has been previously described [22,42,43]. These studies, as well as the present investigation, are based on in vitro modelling microbial shifts by mimicking changes in the oral environment, i.e., factors associated with supragingival and subgingival plaque. The fundamental basis of the development of these studies lies in the assumption that the oral microbial stability can be disrupted by fluctuations in environmental factors, thus bacteria, implicated with for instance disease, are allowed to proliferate and increase in proportions due to changes in the environment, and consequently lead to disease [44].

Gram-negative species have been shown to play a significant role in biofilms grown under disease conditions [42]. The findings presented in this investigation, are in agreement with a previous study [22] and indicate that species, such as *F. nucleatum*, *V. dispar*, *and P. intermedia* increased during peri-implant mucositis and peri-implantitis conditions.

The results herein presented demonstrate that it was possible to disrupt stable communities dominated by *Porphyromonas gingivalis HMT619*, *Prevotella*, *Neisseria mucosa HMT682*, and *Oribacterium*, *Klebsiella*, spp., which were only observed during peri-implantitis conditions. The composition of this in vitro peri-implantitis microbial community is in line with what is reported in previous *in vivo* metagenomics studies [45,46]. In addition, peri-implantitis conditions yielded the highest microbial diversity and quantity. These results are in agreement with previous in vitro and *in vivo* studies [38,47].

The transition towards peri-implantitis exhibited unique genera, such as *Abiotrophia defective HMT389*, *Peptococcus* sp. *HMT167*, *Alloscardovia omnicolens HMT198*, *Catonella morbi HMT165*, *Flavobacteriaceae*, and *Gammaproteobacteria*, spp. peri-implant mucositis. These shifts between health/disease conditions have been previously discussed in a number of clinical studies [46,48,49,50,51], which describe peri-implant mucositis as a pivotal event in disease progression due to the increase in pathogenic bacterial communities. In fact, it has been reported that biofilms associated with peri-implantitis harbour unusual putative periodontal pathogens, such as *Dialister invisus* and *Mitsuokella* spp. HOT 131 [52] or *Filifactor alocis*, which may act as keystone species [53]. Notably, *Prevotella saccharolytica HMT781*, *Prevotella nanceiensis HMT299*, *Filifactor alocis HMT 539*, *Actinomyces odontolyticus HMT 701*, *Leptotrichia buccalis HMT563*, *Leptotrichia*, *Lachnoanaerobaculum*, *Solobacterium moorei HMT678*, *Lachnoanaerobaculum orale HMT082*, *Streptococcus anginosus HMT543*, *Riemerella anatipestifer*, *Campylobacter*, *Selenomonas*, *Neisseria elongate HMT598*, *Neisseriaceae*, *Oribacterium sinus HMT457*, *Capnocytophaga*, *Moraxella osloensis HMT711*, and *Lachnospiraceae [G-8] bacterium HMT500*, spp. were present in both peri-implant mucositis and peri-implantitis conditions, however absent in health conditions.

Previous studies have highlighted the importance of the aerobic Gram-negative bacilli (AGNB) group in peri-implantitis recurrence [54] and disease activity [55], whilst the AGNB groups have also been observed in healthy peri-implant sites [56], the results from this in vitro study highlight the importance of this group in peri-implantitis conditions. Furthermore, recent studies have suggested antibiotic resistance to peri-implantitis biofilms containing species such as *Prevotella intermedia/nigrescens*, *Streptococcus constellatus* [57], and the genera *Klebsiella* [58]. These results encourage further research into the AGNB group in clinical studies.

The use of an in vitro microcosm made up of the whole saliva can aid in the examination of plaque behaviour, ecology, pathology, and for testing potential clinical interventions [17]. However, as with any in vitro system, some limitations may still be present during CDFF modelling. CDFF could be prone to contamination, and it lacks host interactions. Samples must be handled individually, and there is an artificial limit on biofilm development given by maintaining the biofilms at a constant depth or possible mechanical damage of the community induced by high shear forces induced by the PTFE blades/scrapers. Moreover, careful interpretation of the results must be taken, as there is a correlation between the complexity of the microcosm composition and the interpretation of the drawn results [42].

Despite these limitations, our results have shown the complex microcosm shifts and dynamics in the progression from peri-implant health toward peri-implantitis in vitro conditions. We envisage that this standardised model can serve as a basis to investigate future therapies that may be based on altering targeted bacterial interactions, thus leading to disease prevention or cure. Based on the outcomes obtained in this investigation, the CDFF model herein presented can be employed to test in vitro and *in vivo* novel anti-infective therapies for peri-implant mucositis and peri-implantitis.

### 4.4. Characterisation of Biofilm Architecture

Our results suggest that the bacterial morphologies observed (employing both SEM and CLSM) accurately resemble each other. Our findings resemble early studies characterising the microbiota of healthy implants by dark field microscopy, describing cocci bacteria as the main morphotype with a low proportion of spirochetes, fusiform, and curved rods [59,60].

Bacterial qualitative shifts were observed after inducing peri-implant mucositis and then peri-implantitis conditions within the CDFF bioreactor. Specifically, during peri-implant mucositis, an increase in the number of coccoid bacteria, bacilli, and fusiform bacteria was observed. These bacterial shifts have been previously described under conditions associated with gingivitis in vitro [22] and *in vivo* [61]. The transition to peri-implantitis has been previously described by a diversity of morphotypes, specifically the emergence of Gram-negative, motile, anaerobic species, filaments, and fusiform [41,62].

Interestingly, the biofilms appeared to have a final 600 µm of thickness, which was used at the outset of the experiment. This may have been due to mechanical and technical reasons. The mechanical reasons include the dispersal of the biofilm microcolonies while still in the CDFF and the action of the PTFE scrapers inducing high-shear forces in the biofilm. The technical reasons might be inherent to the CLSM processing, which includes the placement of the sample under PBS. This action may cause the fragile parts of the biofilm, which may contain naturally large voids of water channels between the microcolonies, to become dislodged. Therefore, these factors will affect the final thickness measurement of the biofilm. In a clinical scenario, this may be related to the recolonisation of previously treated surfaces with dysbiotic biofilms [63].

An *in vivo* study reported that different surface characteristics appear to harbour different microbial patterns on 4-day supragingival biofilms grown over titanium discs [64]. Machined Ti surfaces exhibited a patched distribution of the bacterial clumps by CLSM analyses, which would be the ideal way to study the spatial distribution of biofilms. Our SEM images were in agreement with the CLSM results where random biofilm clusters were detected, to a similar extent, over machined Ti surfaces. The differences between the present study and the aforementioned study may be due to the immature nature of the biofilms used in that *in vivo* study [64], and the shear forces induced by the PTFE scrapers in the present investigation.

In our study, the early stages of biofilm formation (day 4) were characterised by scattered clumps of biofilms; these patterns seemed to be in line with the patterns in SEM images and followed a patchiness distribution in machined Ti surfaces. Interestingly, immature biofilms often exhibited fewer “red blobs” (i.e., dead cells clump together) than mature biofilms. Then these locations increased following a “patchiness” distribution, and finally, the clumps started to coalesce in mature biofilms, where the number of red clumps decreased but the ones present had a higher volume than the red clumps found in early biofilms.

It was also found that the EPS holds together the viable and non-viable clumps and forms a three-dimensional shape to form the typical biofilm structure [65]. Notably, EPS was in direct contact with the Ti implant surface, forming a layer between the implant surface and the microbes. However, it was not possible to determine the specific thickness of this EPS layer because of the nature of the imaging technique (i.e., submersion of the biofilm in PBS may distort the EPS thickness). In summary, the SEM and CLSM data/images showed a clear transition between a bacterial population that initially (PH) exhibited abundant cocci to then shift to bacilli; filamentous bacteria were observed during PM and PI. Overall, these qualitative results mirror the quantitative/characterisation data derived from CFU/mL and sequencing analyses.

## 5. Conclusions

The CDFF is a useful tool to model complex microbial shifts associated with peri-implantitis communities. A total of 9 phyla, 83 genera, and 156 species were observed across the studied conditions. By employing whole saliva inoculum and a stringent protocol emulating (in vitro) the conditions progressing from peri-implant health, to peri-implantitis, similar bacterial communities were observed on the titanium substrata as shown with structural and characterisation analyses.

## Data Availability

The datasets used and analysed in this study are available from the corresponding author upon request.
